# Evolution of Hybrid LiFi–WiFi Networks: A Survey

**DOI:** 10.3390/s23094252

**Published:** 2023-04-25

**Authors:** Toni Besjedica, Krešimir Fertalj, Vlatko Lipovac, Ivona Zakarija

**Affiliations:** 1 Department of Electrical Engineering and Computer Science, University of Dubrovnik, 20 000 Dubrovnik, Croatia; 2 Department of Applied Computing, Faculty of Electrical Engineering and Computing, University of Zagreb, 10 000 Zagreb, Croatia

**Keywords:** light fidelity (LiFi), wireless fidelity (WiFi), visible light communication (VLC), hybrid network, HWLNet, handover, load balancing

## Abstract

Given the growing number of devices and their need for internet access, researchers are focusing on integrating various network technologies. Concerning indoor wireless services, a promising approach in this regard is to combine light fidelity (LiFi) and wireless fidelity (WiFi) technologies into a hybrid LiFi and WiFi network (HLWNet). Such a network benefits from LiFi’s distinct capability for high-speed data transmission and from the wide radio coverage offered by WiFi technologies. In this paper, we describe the framework for the HWLNet architecture, providing an overview of the handover methods used in HLWNets and presenting the basic architecture of hybrid LiFi/WiFi networks, optimization of cell deployment, relevant modulation schemes, illumination constraints, and backhaul device design. The survey also reviews the performance and recent achievements of HLWNets compared to legacy networks with an emphasis on signal to noise and interference ratio (SINR), spectral and power efficiency, and quality of service (QoS). In addition, user behaviour is discussed, considering interference in a LiFi channel is due to user movement, handover frequency, and load balancing. Furthermore, recent advances in indoor positioning and the security of hybrid networks are presented, and finally, directions of the hybrid network’s evolution in the foreseeable future are discussed.

## 1. Introduction

Short-range wireless technologies are becoming crucial in fifth generation (5G) networking. It is predicted that there will be 17 billion mobile devices and WiFi hotspots by the end of 2030, in contrast to 549 million hotspots in 2022 [1]. Because of radio frequency (RF) limitations, WiFi access points must be densely deployed, which in return results in the congestion of available channels. Further, the demand for mobile traffic is exponentially increasing, for e.g., from 2017 to 2022, it increased by seven times to about 77.5 Exabyte monthly.

To solve these problems with respect to the RF spectrum, researchers are employing technologies with extremely high frequencies such as light fidelity LiFi [2], which uses light as an information carrier and exploits the vast optical spectrum of about 300 THz. Its biggest advantage is the ability to integrate LiFi access points into the existing light infrastructure, which enables Access Points (APs) to serve a dual purpose (illumination and communication). In recent research of light-emitting diodes (LEDs), it was shown that a single LiFi AP could achieve data rates of 10 Gbps [3].

Furthermore, LiFi offers:

- license-free spectrum;
- usage in environments unfavourable to the RF spectrum (hospitals, underwater, LNG ships);
- physically secure channels (the light does pass through walls).
- Still, there are some limitations:
- LiFi APs have a short range of coverage (of about couple metres around a single AP);
- its physical property (which is its biggest security advantage) becomes its worst drawback, because light is prone to obstructions and therefore to the loss of signal.

**Figure 1:**
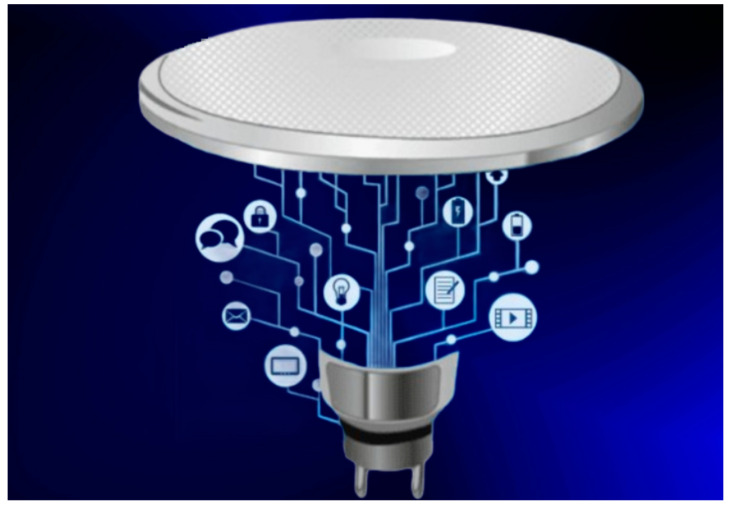
Vision of future LiFi network possibilities.

The hybrid LiFi–WiFi networks (HLWNet) were first mentioned in the paper of Rahaim et al. in 2011 [4]. Later on, the research was extended to the integration of LiFi and femtocells (with a small low-power cellular base station designed for home use, with DSL from telecom service providers and integrated WiFi AP and router capabilities), which in fact showed that such hybrid networks achieved better performances than standalone ones [5]. Figure 1 shows the representation of LiFi possibilities in its integration with 5G technologies. For example, outside of buildings or structures, users can connect to the internet via satellites or 5G LTE stations and inside, they can connect to HWLNet.

To the best of the authors’ knowledge, no other work in the literature provides a comprehensive review of the recent HWLNet advances and research challenges, specifically with regard to handover, load balancing, positioning, and security models for HWLNets, which is a novelty and a contribution of this manuscript.

The contributions of this paper are as follows:

- we present a framework for HWLNet analysis (architecture, cell deployment, modulation scheme, illumination, and design of backhaul devices);
- systematic review of the performance metrics of HWLNet is provided;
- we discuss user behaviour models and their effects on HWLNet performance;
- thorough analysis of HWLNet handover models and load balancing is presented;
- we give an introduction of HWLNet to the Internet of Things (IoT) with embassies on physical security;
- finally, we discuss future implementations of HWLNets.

The structure of this paper is the following:

In the Section 2, we present the framework of the HWLNet architecture, while in the Section 3, we review the metrics of the hybrid networks. In the Section 4, we discuss the effect of user behaviour and then present handover models and load balancing. Furthermore, in the Section 6, we present the physical security of HWLNets and their introduction into IoT. Finally, the upcoming future implementation issues, challenges, and advancements of HWLNets are discussed in the Section 7, followed by the conclusions in the Section 8.

## 2. Framework for HWLNet

In this paper, a framework is introduced with aspects on: network architecture cell deployment, modulation scheme, algorithms, and design of backhaul devices. The goal is to provide directions for the HWLNet design.

### 2.1. Architecture

**Figure 2:**
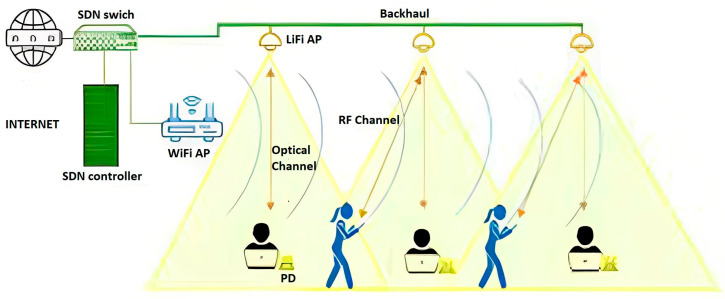
SDN-enabled HWLNet.

There are two basic ways to incorporate LiFi into a WiFi network: autonomous and centralized. In the autonomous method, the WiFi network is extended with LiFi APs to a degree to which a user can freely choose any type of AP from the two technologies. Even though this approach is less complicated in terms of network management, it offers low performance in terms of user handover and connectivity. A centralized approach offers a more controllable network via a central control unit. This enables us to optimize routing tables and resource allocation via a software defined networking controller (SDN). One type of this network uses an SDN-enabled switch to connect LiFi and WiFi APs with the use of SDN agents, which in turn send information to the SDN controller, which uses given information to make decisions on packet routing and user handover, as shown in Figure 2.

To the best of our knowledge, a fully operational SDN for real-world applications has not yet been developed. There are many models and research projects such as 5G-CLARITY that simulate the use of SDN controller, but the development of HWLNet is still in its experimental phase [6].

### 2.2. Cell Deployment

In contrast to WiFi technology, wherein signal can reach indoor users up to 50 m [7] and a few hundred metres [7] in an open area, LiFi suffers from a short-range coverage due to light propagation properties as well as the currently available LED technology level. Therefore, it is vital for LiFi AP to be placed in a way that optimizes its deployment to achieve high-quality network performance. Usually, LiFi APs are integrated into ceiling lamps, which are susceptible to different constraints such as room shape and size, number of windows, height from floor to ceiling, and other factors. There are three main cell deployment models: hexagon, matrix, and Poisson point process (PPP), described as follows:

- hexagon deployment is borrowed from cellular networks, and it provides the highest signal to interference-plus-noise ratio (SINR) for LiFi [8]. This type of lamp arrangement is not common in real world applications, so it is used as an ideal model for testing;
- matrix deployment is the most common model used in research because of its simplicity. Research shows that it comes very close to hexagon in terms of SINR;
- PPP is primarily used for simulation of random distribution of APs. It is based on the Poisson equation and has the worst SINR [8].

**Figure 3:**
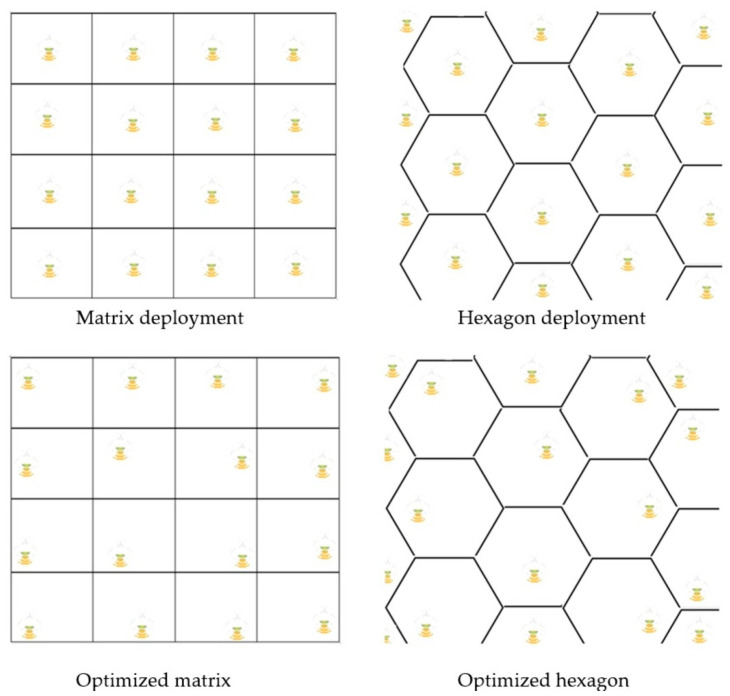
Different types of LiFi AP deployments.

The distance between APs in LiFi networks is a trade-off between the handover rate and coverage. There were several studies on matrix deployment [9], which concluded that handover with an average speed of users is best at a three-metre distance between APs. Another study [8] used gradient projection to find the optimized distribution of APs. These distribution types are shown in Figure 3 and show that optimized types can improve the system throughput by up to 70% [10]. It must be taken into account that WiFi APs affect the placement of users, therefore influence the performance of HWLNets. Research has shown that matrix deployment of WiFi APs can increase the system throughput by 20% compared to that of traditional deployment [10].

### 2.3. Modulation Schemes

**Figure 4:**
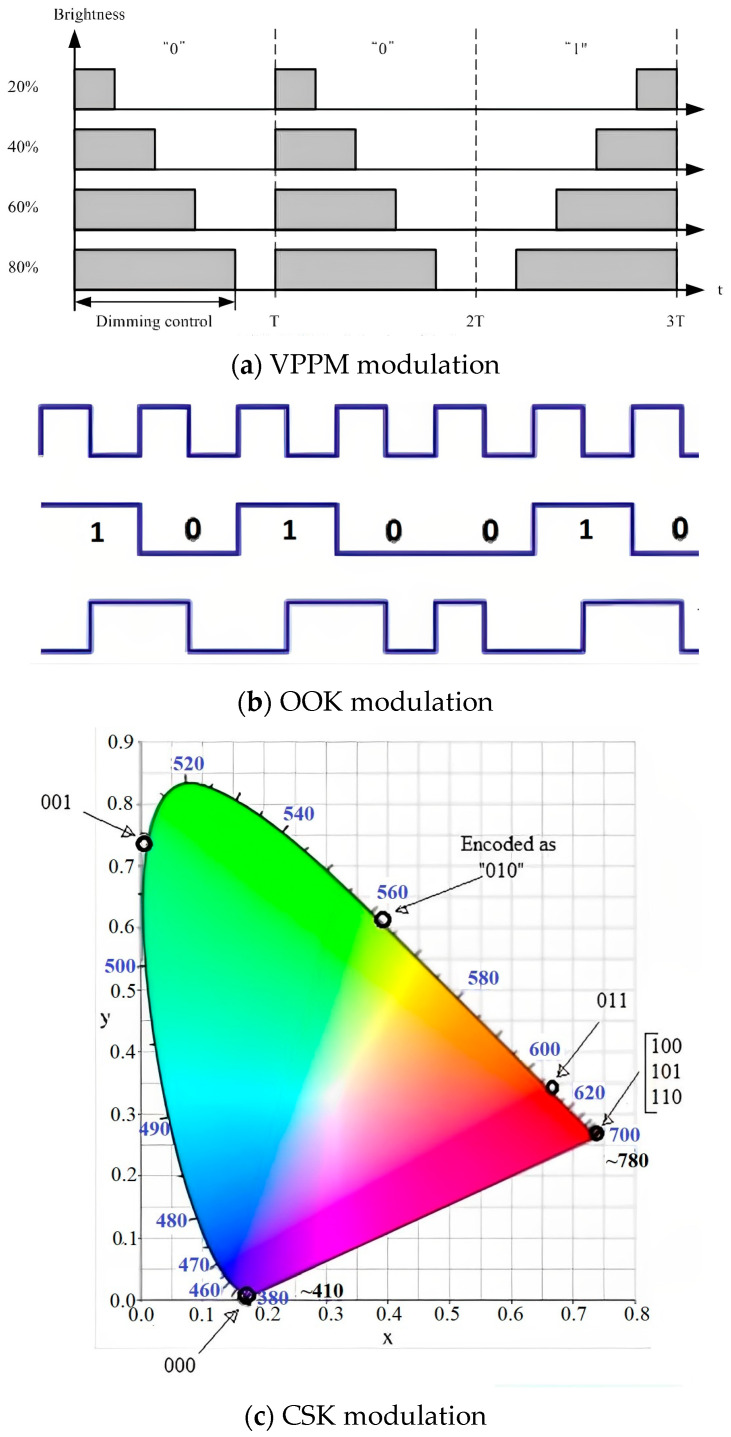
Representation of VPPM and OOK and CSK modulations: (**a**) VPPM, (**b**) OOK, and (**c**) CSK.

LiFi standards for modulation schemes are defined by IEEE 802.15.7 [11] with VPPM/OOK/CSK modulation techniques presented in Figure 4. The Variable Pulse Position Modulation (VPPM) is used to achieve variable range and constant data rate by adjusting the pulse width. This modulation’s largest benefit is protection from intra frame flicker. This is achieved by constant pulse amplitude and variable pulse width to control dimming. Logic ‘0’ and ‘1’ are mapped using positive pulse at the beginning followed by negative pulse and negative pulse at the beginning followed by positive pulse, respectively. This type of modulation is efficient only if the time span that contains pulses is long enough to distinguish pulse positions.

In the On Off Keying modulation (OOK), data are represented by turning the LED diode on and off. In simple terms, light states such as ‘ON’ constitutes logic ‘1’ and ‘OFF’ constitutes logic ‘0’. OOK uses the Manchester code to represent digital information with edge transition concepts, where low to high represent logic ‘1’ and vice versa for logic ‘0’. This technique achieves a variable data rate at constant range with the insertion of compensation time.

Colour Shift Keying modulation (CSK) is used to represent bits of information in the form of different colour wavelengths. Red, green, and blue LEDs are used on the transmit side to produce different colours with different wavelengths to encode the information bits. The diagram maps different wavelengths denoted as ‘blue’ with respective colour counterparts. This type of modulation is most expensive to manufacture because it uses a complex design of transmitters and receivers, which is the only drawback of this scheme. The advantages of CSK far outnumber the disadvantage and make this modulation very popular. The most notable advantages are:

- colour co-ordinates represent information, which in turn is represented in the shape of binary codes for programming clarity;
- information is rendered by the light’s colour; amplitude is maintained, thus the sum of the average light source power is constant;
- CSK assists in realizing variable high bit rate because it uses high order modulation support such as 4-CSK, 8-CSK, and 16-CSK.

**Figure 5:**
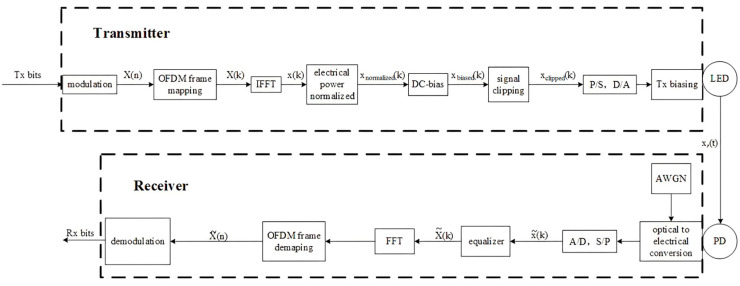
DCO-OFDM modulation.

Another LiFi standard is ITU-T G.vlc with the DCO-OFDM modulation technique, as presented in Figure 5. The direct current-biased optical orthogonal frequency division multiplexing (DCO-OFDM) technique is applied to LOS (line of sight) and non-LOS models to achieve high-speed data transmission and maximizes the channel capacity by applying power and bit loading. This DCO version of OFDM is used only in VLC because of the need for real-valued nonnegative symbols. This is achieved by applying Hermitian symmetry to the information frame before the inverse fast Fourier transformation (IFFT). The information is transmitted by modulating the amplitude of the LED signal at a sub-carrier frequency. The sub-carrier frequencies are selected to be orthogonal to each other, so they do not mutually interfere. This technique helps in reducing the interference between the sub-carriers and increasing the system’s spectral efficiency. However, the LED-based transmitter has a non-linear response, which distorts the transmitted signal. This distortion can be reduced by introducing a direct current (DC) offset in the signal to linearize the LED’s response and improve the system’s performance. On the receiver side, a PD receives the modulated signal and then converts the optical signal into the electrical one. The received signal is then demodulated using fast Fourier transform (FFT) to recover the original signal. DCO-OFDM has several advantages over other modulation techniques for VLC systems. It is a simple and efficient technique that can achieve high data rates with low complexity. It does not require any additional hardware or complex signal processing algorithms, which makes it a cost-effective solution for VLC systems.

Researchers [12] have discovered the optimized scheme for the distribution of power and constellation sizes on different subcarriers, which is achieved by maximizing the minimum Euclidean distance of the constellations.

These modulation schemes are used as a part of multiple access standards for LiFi. Carrier sense multiple access/collision avoidance (CSMA/CA) and time-division multiple access (TDMA) are now for only LiFi standards. LiFi, unlike WiFi, uses one channel for downlink (visible light) and another for uplink (infrared), which in turn causes collisions if CSMA/CA is used. Authors in [13] found an option to broadcast a channel busy tone to reduce collision. The better option is to use a different multiple access standard all together. TDMA achieves better power consumption and bandwidth utilization. The orthogonal frequency-division multiple access (OFDMA) and non-orthogonal multiple access (NOMA) are other two multiple access schemes researched for use in a LiFi environment. These schemes require a very complex system because of the coordination of resource assignment. OFDMA uses time-frequency resource blocks to support concurrent transmission, and NOMA groups users by power levels and channel condition rather than time-frequency in OFDMA. NOMA has better performance only if there is a large difference in the channel conditions. Authors in [12] found that the angle of LiFi transmitters also has high impact on NOMA.

Data rates for LiFi modulation are as follows [14]:

- from 10 kbps to 100 Mbps for OOK and VPPM;
- from 12 Mbps to 96 Mbps for CSK.

### 2.4. Illumination

In order to comply with the illumination regulations given by the international organizations for standardization (ISO), which specifies 300 lux to max 1500 lux illuminance for office work, researchers [15] have derived a model for optimal illuminance.

**Figure 6:**
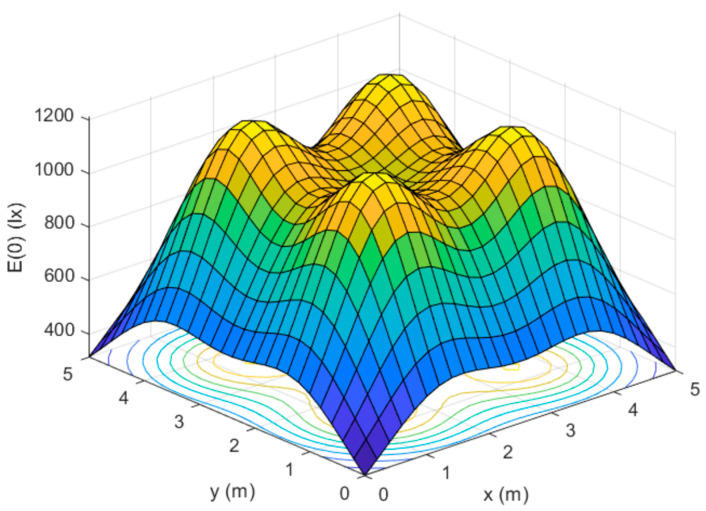
Illuminance distribution in a room.

A model for optimal distribution of LiFi APs is given in Figure 6, where the location and strength of LED lights’ output are such that the corners of a room have 300 lx and max. illuminance does not exceed 1200 lx, which is well within the parameters of the ISO. Developing LiFi networks with illumination requirements involves positioning LEDs in optimal locations in a room, determining their orientation towards users and each other, developing emission patterns, and determining max. and min. output powers and colour temperatures. A paper [16] proposes a power allocation scheme to obtain the max. number of users under certain colour temperatures.

The obtained results show that high data rates are in direct coalition with high-colour temperatures due to LED properties, since an increase in energy level provides more visible light, thus implying higher throughput. Another study [17] discovered that maximizing the throughput of data and higher colour temperatures caused an increase in LED flickering. This study suggests that a control mechanism should be in place to prevent eye damage and injuries.

### 2.5. Backhaul Devices

These devices form the backbone of HWLNets, and their main goal is to accommodate a relatively large number of APs and differences in network (LiFi and WiFi users) demand for high network capacity, such as one LiFi AP providing data rates in Gbps. There is a vast number of technologies that are being researched to find an optimal backhaul solution. All of these can be classified as wireless or wired backhaul.

**Figure 7:**
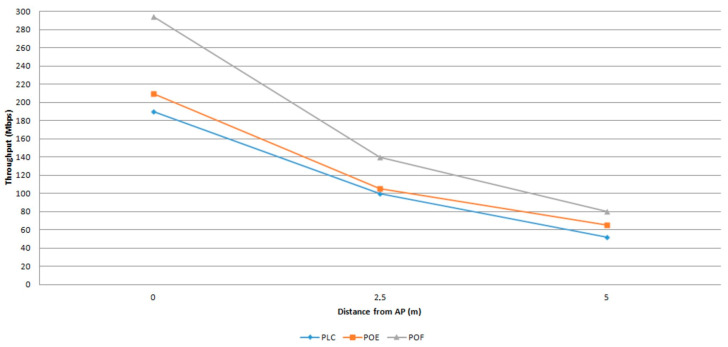
User’s achieved data rates in different backhaul configurations.

Wired backhaul such as power line communication (PLC) can provide stabile connection at low cost using existing electrical wires proposed in [18]. The latest developments [19] show that the hybrid PLC–VLC structure can handle multiple users with backhaul data rates up to 1 Gbps. Another wired backhaul use is provided by the power over Ethernet (POE) devices. Moreover, dual-hop relay transmission with a series of alternating POE and VLC devices can achieve backhaul data rates of 1 Gbps [20]. Finally, plastic optic fibre (POF) can be used for backhauling [21] with frequency division multiplexing to transmit signals to LiFi APs with backhaul data rates up to 15.7 Gbps [22]. Figure 7 presents the maximum user throughput at given distances of 0 m, 2.5 m, and 5 m from a LiFi AP for different backhaul technologies. The achieved throughput differs from the data rates of backhaul solutions due to physical differences in the LED transmitters used in the studies and due to different modulation schemes.

Wireless solutions are more complex and have higher costs but offer more flexibility in installation. Recent developments [23] have exhibited backhaul devices on the base of a millimetre wave (mmWave) with point-to-point connection. Another study [24] uses VLC-base as a backhaul device with in-band full-duplex for both access and backhaul. Finally, VLC and infrared can be used for backhaul as well [25] with a distinction of inter network between the backhaul devices and APs.

The authors of this paper believe that the allocation of LiFi–WiFi resources can be best used if they share the same backbone. For now, wired backhaul devices have shown greater reliability with lower costs compared to their wireless counterparts. Therefore, future HWLNets should use POF technology for intra-networking of backhaul devices accompanied with POE devices to form a more reliable and flexible backhaul. The use of wireless backhaul technology should be investigated further as it is the future of indoor networking.

## 3. Performance of HWLNets

The HWLNets performance is measured with SINR coverage, spectral efficiency, energy efficiency, network capacity, and quality of service (QoS). This section provides an overview of these metrics.

### 3.1. SINR Coverage

The SINR for each user must be above a certain margin to provide a stable connection. RF devices use omnidirectional antennas and the signal is dependent on distance and obstacles. For VLC, user’s orientation has to be considered; if the wave-front is parallel to an interface, signal power is at maximum, otherwise if the user’s equipment is beyond the line of sight of the LED transmitters, no signal will be received. Because of light’s physical property, user orientation can influence the coverage area of APs [26]. There are two basic ways of solving the coverage problem:

- using a large photo diode (PD), which in turn expands a user’s ability to receive more light but also more interference;
- using multiple narrow PDs with an angle diversity receiver (ADR) as shown in [27].

Researchers have demonstrated that HWLNets can improve SINR over standalone networks with the use of single PD receivers and an angle of view below 45° [28].

**Figure 8:**
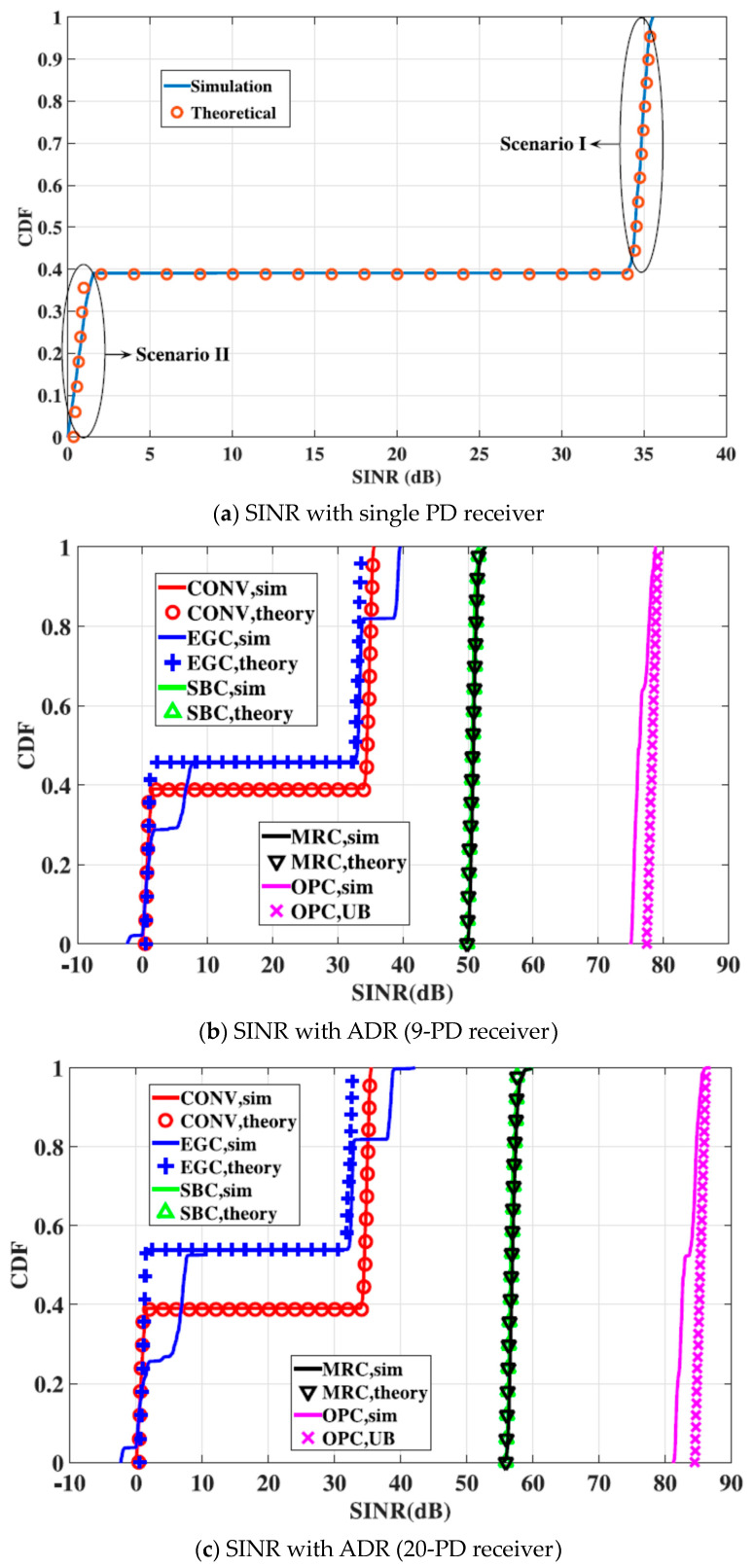
CDF performance of received SINR for different PD configurations [27].

In [27], researchers derive the cumulative distribution function (CDF) of received SINR to determine performance. This study uses a simplified non-line-of-sight (NLOS) model because the conventional model is unmanageable. In Figure 8a, two categories of a single PD receiver, Scenario I and Scenario II, are presented. Both depend on the user’s position and are statistically analysed. In the first scenario, all users are located around the cell centre with no LOS interference, whereas in the second scenario, LOS interference is present.

Researchers [27] have shown that in the Scenario I, with limited field of view (FOV), a PD LOS neighbouring interference can be rejected so that NLOS remains, thus resulting in high SINR. At the cell edge, low SINR is present, as shown in Scenario II, due to LOS.

Multiple PDs [27] use signal-combining schemes such as select best combining (SBC), equal gain combining (EGC), maximum ratio combining (MRC), and optimum combining (OPC). Tools and analyses of PD receivers can be extrapolated for use in angle diversity receivers (ADRs) with the assumption that each PD points to a different location (no overlap) and can establish one LOS link with AP.

Combining schemes for ADR with 9 or 20 PDs, as presented in Figure 8b,c [27], are as follows:

- SBC: user in centre selects upward pointing PD to get the best SINR;
- EGC: user’s received signal is similar to SBC in the centre of the cell due to the fact that only one PD can receive the LOS signal. In this scheme, weights of PDs are the same and NLOS will increase with the increase in PD numbers;
- MRC: different PDs have different weights by their SINR. This model can establish two LOS links with APs;
- OPC: LOS signals are captured by two PDs that establish LOS to APs similarly to MRC.

Authors in [27] have demonstrated that SINR performance with a nine-PD receiver is significantly improved compared to that of a single PD due to SBC choosing PD that is best located to obtain high channel gain. This scheme has a narrow FOV and no LOS interference. Compared to SBC and MRC, OPC has the best SINR performance because it uses an interference-plus-noise correlation matrix to generate optimized weights, thus suppressing NLOS interference and achieving 20 dB improvement. For the 20-PD receiver, SINR performance of EGC is similar to the nine-PD EGC. In SBC and MRC, the 20-PD receiver has 5 dB improvement, and in OPC, with even narrower FOV and higher concentration of PDs, 20-PD can receive stronger light signals, resulting in better SINR performance over the nine-PD receiver.

### 3.2. Spectral Efficiency

Spectral efficiency is an important consideration in designing and implementing visible light communication (VLC) systems, particularly in hybrid LiFi–WiFi networks that use dual-mode devices to transmit data over radio frequency (RF) and optical channels. In such networks, maximizing spectral efficiency is crucial to ensure that the available bandwidth is used as efficiently as possible. One approach to achieving high spectral efficiency in hybrid LiFi–WiFi networks is using differential carrier-offset electronic frequency division multiplexing (DCO-EFDM) modulation. This technique enables the simultaneous transmission of multiple data streams over a single optical carrier frequency, significantly increasing data throughput.

DCO-EFDM modulation uses multiple subcarriers to modulate the amplitude and phase of the optical carrier signal. The subcarriers are closely spaced in frequency, with a small offset between each subcarrier. This allows for a higher spectral efficiency, as multiple data streams can be transmitted over the same optical carrier frequency without interfering with each other.

Spectral efficiency can be interpreted as the quantity of data transmitted over a given spectrum or bandwidth with minimal transmission errors. The VLC system [29] was constructed with 4.85 bit/s/Hz spectral efficiency based on carrier less amplitude and phase modulation (CAP). Another paper denotes the use of generalized spatial modulation with dimming control to achieve results over 10 bit/s/Hz. Improvement in spectral efficiency was made with DCO-EFDM in [30] with adaptive bit load and a speed of 15.73 Gbps. HWLNets allow users to be moved from one to another network depending on SINR values, which in terms will increase spectral efficiency by up to 30% [5].

Area spectral efficiency is defined to compare LiFi and WiFi systems by measuring the sums of max. average data rates of bandwidth on an area. Researchers [31] have determined that LiFi has 10-times more ASE than WiFi and hybrid networks will have at least 2-times more ASE than standalone networks. It has been found that small obstacles can actually prove beneficial to VLC because they block more interference than the desired wavelength [32].

In summary, spectral efficiency is a critical consideration in designing hybrid LiFi–WiFi networks, and DCO-EFDM modulation is an effective technique for achieving high spectral efficiency in VLC systems. By leveraging the strengths of both VLC and WiFi technologies, hybrid networks can provide high-speed, reliable data transmission in a wide range of environments.

### 3.3. Energy Efficiency

With the increase of energy prices, this metric has become one of the key factions in determining the profitability of investments in HWLNets. The consumption of energy rises with the number of APs, so there is a trade-off between energy and spectral efficiency. One of the key factors that can impact energy efficiency in these systems is the choice of modulation technique. Orthogonal frequency division multiplexing (OFDM) is a popular modulation technique used in both RF and VLC systems, and its energy efficiency can be improved by the use of specific OFDM variants.

One OFDM variant that can improve energy efficiency in hybrid LiFi–WiFi networks is asymmetrically clipped optical OFDM (ACO-OFDM). ACO-OFDM [33] uses asymmetric clipping to reduce the peak-to-average power ratio of the signal, which reduces the amount of energy required to transmit the signal. This technique can be particularly useful in VLC systems, where high peak-to-average power ratios can lead to significant energy losses.

Another OFDM variant that can improve energy efficiency in hybrid LiFi–WiFi networks is DC-biased optical OFDM (DCO-OFDM) [33]. DCO-OFDM uses differential carrier-offset modulation to achieve high spectral efficiency, allowing multiple data streams to be transmitted simultaneously over a single optical carrier frequency. This increases the amount of data that can be transmitted per unit of bandwidth, which can reduce the overall energy consumption required to transmit the data.

OFDM-based systems such as ACO-OFDM have more energy efficiency than for example DCO-OFDM for low spectral efficiency (2 bit/s/Hz) [32].

In addition to these specific OFDM variants, there are other techniques that can be used to improve energy efficiency in hybrid LiFi–WiFi networks. One approach is to use adaptive modulation and coding, which adjusts the modulation scheme and coding rate in real-time based on channel conditions. This can help to ensure that the system is using the most energy-efficient modulation technique for the current channel conditions, reducing energy consumption.

Another approach is to use power allocation techniques, which adjust the power level of each subcarrier in the OFDM signal based on the channel conditions. This can help to ensure that the system is using the minimum amount of energy required to transmit the signal while still maintaining the required quality of service.

HWLNet system can optimize energy efficiency by granting bandwidth and power allocation to certain parts of the system [34] where more power is needed, thus restricting power to other parts and having efficiency improve by about 75% in contrast to the traditional WiFi system while minimizing energy consumption and reducing environmental impact.

### 3.4. Network Capacity and QoS

In wireless networks, capacity is a measure of the maximum data rate of the network with regard to bit error ratio. In HWLNets, the network capacity can be increased by increasing the available bandwidth. This can be achieved using wider optical channels in the VLC component and higher frequency bands in the RF component. HWLNets can improve network performance by taking excess traffic or providing certain users high data rates for bandwidth-consuming applications such as 4K video streaming. This especially helps large and dense WiFi networks. On the other hand, WiFi can provide stable connections to areas that cannot have decent LiFi coverage [32]. This type of network has been extensively researched in the last couple of years, focusing on load balancing which will be elaborated on in Section 5.

However, increasing the bandwidth can also lead to higher signal attenuation and interferences, affecting the quality of service (QoS). Packet throughput, loss ratio, latency, and jitter, unlike capacity (part of the physical network layer), are measured on the network layer where support for applications with diverse QoS requirements is necessary. To ensure both network capacity and QoS, HWLNets can implement several techniques. One technique is to use multiple-input multiple-output (MIMO) [35] technology, which uses multiple antennas to increase the network capacity and improve the signal quality. Another technique uses adaptive modulation and coding [36], which adjusts the modulation scheme and coding rate in real-time based on channel conditions. This can help ensure that the system uses the most efficient modulation technique for the current channel conditions, which can improve both network capacity and QoS.

HWLNets can also use advanced transmission protocols, such as hybrid automatic repeat request (HARQ), to improve the QoS of the network [37]. HARQ is a retransmission-based protocol that retransmits only the lost data packets, reducing the number of retransmitted packets and the associated latency.

Some applications such as 4K video streaming and 3D holographic require high data rates and others such as remote surgery or automated guidance for vehicles (AGV) need low latency [38]. With the growing number of user equipment requiring more bandwidth from WiFi APs and thus increasing the average latency and jitter, one of the possible improvements that would be cost-effective and solve the demand for high packet rate would be implementation of LiFi APs in hybrid software-controlled networks [39].

User experience is another requirement that is significant to any network. This measures the average user satisfaction with a given service and thus determines the need for a specific type of network. Jain’s fairness index, used for evaluating the fairness of resource allocation among traffic flows, can be used to establish a network suitable to a vast number of users with different application requirements. Fairness schemes in HWLNets are also discussed in Section 5.

## 4. The Effect of User Behaviour on HWLNets Performance

Networks, in general, are affected by users. For traditional RF networks, the number of users and their demand for high data rates due to bandwidth consuming applications affects the speed and usability of the network. In VLC, LiFi standalone network, or HWLNets, factors that influence performance due to light’s physical properties are also user’s mobility and device orientation following light blockages. All these factors are commonly known by the term user behaviour. This section provides a brief overview of user behaviour models.

### 4.1. User Mobility

User mobility models are arranged in the following categories according to movement memory or restriction:

- random models;
- dependency models;
- geographic models.

The behaviour of users indoors is very complex and variable, with many different models trying to describe user movement at certain speeds [40]. One model tries to mimic user movement by defining rules a specific path which a user would follow while moving. These models are established based on an emphasis that each user will behave in a certain way in a specially designed environment, which in practice is impossible. Another type of model is random waypoint (RWP) [41] that simulates the movement of users in winding lines, touching some randomly allocated markers (so called waypoints) as they go. The variable of speed in this model is constant, and the user always moves forward. Distances in this model are short, so the speed of users could be calculated as constant over a short period of time [42]. This model is used for determining optimal locations for APs [43] in HWLNets as it gives increased mobility prediction but longer task execution time and high energy consumption. The latest research in the field of mobility-aware computational offloading is presented in [44], which is applicable to hybrid networks.

Task offloading uses SDN-based models [44] to handle network requirements such as load balancing and handover but does not consider the user’s geographic location or mobility. This can be solved by combining SDN and Markov chain to predict future network conditions. The complexity of computation in Markovian models makes prediction difficult to obtain with a high degree of accuracy. The Leavy Walk [44] model uses randomness as a function to predict user’s location but cannot provide an optimal solution. Self-similar Least Action Walk (SLAW) [44] uses walk behaviour to predict the user’s next location. This model has turned out to be very good in predicting user patterns if the delay can be tolerated; however, in a delay-sensitive environment, its efficiency is yet to be tested.

Another approach is the Headlight model (HLM) [44], which uses statistical analysis of user mobility records to forecast future user locations. HLM picks up zones adjacent to the direction of movement, such as the headlight of a moving vehicle. Unfortunately, HLM has no global view of network resources, and predictions are inadequate.

Machine learning models [44] for solving user mobility problems use Deep Neural Networks (DNN) or Reinforcement Learning (RL). These algorithms have high time complexity due to the increased number of users present and the fact that they use linear mapping with some entry parameters and features. Input data for learning models are not linear, thus adding complexity to predict user movement with these models.

Evolutionary algorithms such as Genetic algorithm (GA), particle swarm optimization (PSO), and Ant Colony Optimization (ACO) can also be used to describe the behaviour of users because these models work very well in multi-objective scenarios [44]. GA and PSO can be used to optimize the placement of nodes based on criteria such as signal strength, coverage, and connectivity. ACO, a metaheuristic algorithm inspired by the behaviour of ant colonies searching for food, can be used to mark the user’s movement through a problem space.

The above-mentioned models have straight movement-based meaning prediction of user behaviour and are limited by movement in line, whereas in real-world scenarios, users can move in loops or elliptical curves. Solving user mobility can be achieved by new models that will consider dynamic mobility patterns. Current mobility models used in HWLNets underline that moving users are served with WiFi rather than LiFi to keep certain thresholds in QoS [45].

### 4.2. Device Orientation following Light Blockades

**Figure 9:**
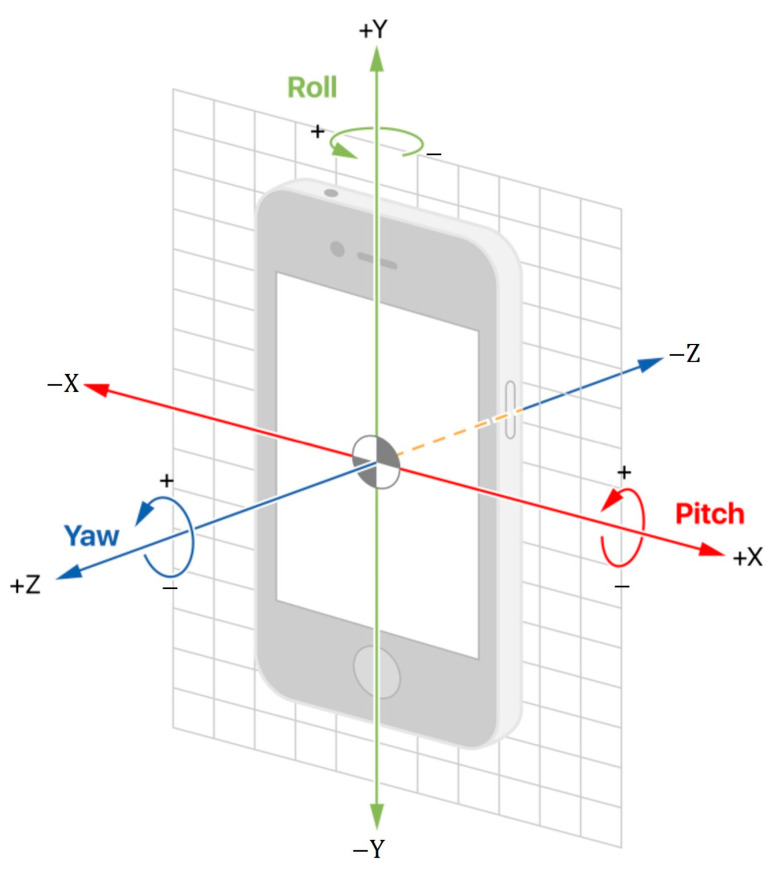
Axial rotation of smartphone.

User equipment for LiFi communication must have a type of photodiode (PD) capable of detecting an interpreting light. PDs are small and have a narrow field of view, therefore they cannot detect light in all angles. Data transfer rates and QoS service in VLC are measured in luminosity in contrast to direction of the equipment towards the source of emission. This is a very complex issue that was first researched in [46] with the assumption that randomly oriented devices will follow Euler’s rotation theorem. Euler’s rotation theorem states that in three-dimensional space, any displacement of a rigid body such that a point on the rigid body remains fixed is equivalent to a single rotation about some axis that runs through the fixed point. This type of movement can be best described by formulating three axial rotations in the Cartesian coordinate system, as shown in Figure 9. The researchers obtained rotational data from gyroscopes in smartphones [47], which in turn revealed that while fixed smartphones have Laplace distribution of rotation, smartphones in motion obey Gaussian distribution. Unconstrained smartphone orientation models are presented in [48] with the distinction that here the polar angle of the smartphone best suits Laplace distribution.

Orientation changes can be researched from a different view such as in [49] using data measurements. In this research, the time over which the channel-gain-values correlation coefficient drops below a predefined threshold in random orientation is in milliseconds. For VLC systems where delay is in nanoseconds, this means that rotation can be managed as slowly-varying channels. The problems with recurring handovers in HWLNets are best described in [50] using a device orientation model with RPW, which comes up with a practical framework for VLCs. This model is also used to describe load balancing and concurrent asset allocation for users [51].

Blockage models are incorporated with a RWP model due to the fact that besides walls and objects such as furniture, humans carrying a device can also block light from reaching a PD. This type of blockage can be partial or full depending on the position of the user and device in accordance with LiFi AP and the number of users present in a room. In this model, humans are represented in a cylinder form [52]. These data have been statistically modelled [53] with a basepoint being on a frequency of blockades and its duration. There are a couple of hardware modifications that can be made to alleviate blockades such as LED transmitters with a wide angle to expand light broadcasting but with more interference [54] and omnidirectional receivers with PDs on each side [55]. In HWLNets, another option is for the SND controller (requires vertical handover) to switch users in and out of different networks due to blockades, thus solving this comprehensive problem.

## 5. Handover and Load Balancing in HWLNets

The transfer of users to a different type of network in hybrid systems is called handover. There are two types of handovers: horizontal (HHO) and vertical (VHO). The main difference is in terms of the technology that would serve the users as they advance through the network. In HHO, users are being served by APs of the same technology; for example, if a user is moving in and out of different rooms covered with WiFi, APs thus reconnect the user to a network via different APs. In VHO, the user changes the type of technology used to access a network. In HWLNets, if the user is stationary in one room, they are served with LiFi AP, and when they decide to move, they will exit out of the FOV of the LiFi transmitter and will be reconnected to the network by the use of RF to the nearest WiFi AP.

A lot of research has been done on this topic concerning heterogeneous networks (HetNets) [56] with different types of handover models used: received signal-strength (RSS), load balance, and energy-save. In HWLNets, the handover process is complex, and in this section, we will explain and review the handover models in LiFi and WiFi environments and also vertical transfer between the two.

### 5.1. HHO

Because of the physical property of light and technological ability of LiFi transmitters, AP covers up to three metres in diameter, thus making LiFi susceptible to frequent handovers even if the users move slowly. The data rate of LiFi is also affected by the PD in the user devices who can change orientation and affect the connectivity and in turn lead to handovers. In VLC, especially LiFi networks handover is a considerable factor in terms of QoS and user fairness. There are two major restrictions in distances between APs:

- short distance leads to high spectral efficiency;
- longer distance decreases the number of handovers.

A study was made by [9], in which the researcher proves that an ideal coverage for a single AP is two to eight m^2^, taking into account the number of users and handover overhead. In this model, the overlap of light was pronounced due to the relatively close proximity of LiFi APs, and further study was carried out [57] that simulated the handover for non-overlapping APs. In this research, authors proposed the use of two handover schemes:

- soft handover when distances between AP are great, so there is little or no overlapping. User equipment starts to communicate and establishes connection with the AP that has better QoS requirements before breaking connection with the old AP;
- hard handover for short AP distances. User equipment breaks connection with AP and only than establishes connection with the new AP.

In another study [26], user equipment rotation and speed were analysed for handover, resulting in frequent handovers when equipment was angled between 60 and 80 degrees.

The above-mentioned models represent stationary or slow-moving users. Handover rates of fast-moving users are extremely high, so a new model had to be made. One such is handover skipping [58] for unlinked APs. This novel topology-aware scheme proposes that a user should skip adjacent APs while moving in a predetermined vector. This can only be accomplished if a network controller has knowledge of the user’s movement in such way that it can predict behaviour to an extent that a user will be fed by specific APs to diminish frequent handovers and to maintain a certain level of QoS. Such schemes relay on static network topology, which LiFi is anything but. A problem with positioning methods of users via GPS is that it is not pin point accurate, especially in buildings, which would in turn need a forecast model of sort that would rely on previous user movements combined with machine learning technology such as fuzzy logic. Further, sending this information back to APs is required, thus an RSS handover model was researched [59] based on the rate of change in RSS to determine a user’s movement in relation to AP. This model uses average change rates in RSS to make handover possible since this method has no extra feedbacks and does not need to know network topology. This research showed that RSS handover can improve network data rates by about 30% over trajectory-based handover methods.

### 5.2. VHO

**Figure 10:**
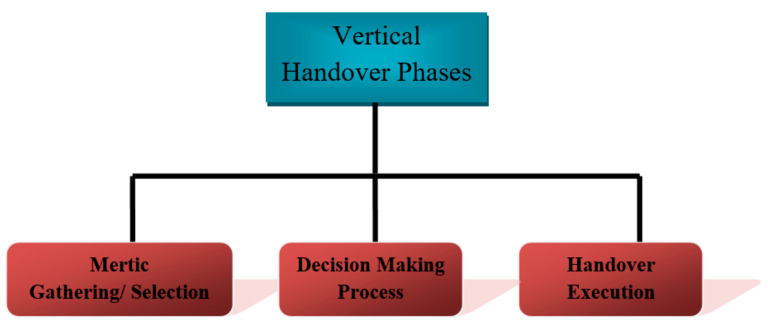
Phases in vertical handover model.

When there is evident loss of connection in HWLNets, a user is transferred from LiFi to WiFi, evidently changing the technology used to receive data, thus this is called vertical handover. Its phases are shown in Figure 10.

There are two main reasons why this would happen:

- light is blocked by an object;
- user equipment is oriented in such a way that LoS with AP is impossible.

VHO probability has been researched [60], resulting in apparent reciprocity in the number of handovers and their delay. One scheme [61] proposes the use of Markov decision process to determine the implementation of VHO by using the queue length of WiFi and the channel conditions of LiFi as metrics. Another scheme [62] uses interruption duration, message size, and access delay as metrics recorded on user devices to make VHO. A third scheme [63] was developed using delay, queue length, data rate, and traffic preferences. These models relay on channel and traffic conditions to determine the best time to execute handover. User mobility and overhead also play a key role in the handover process and have different constraints than the above-mentioned models.

### 5.3. HHO and VHO in HWLNets

HWLNets rely on handover between WiFi and LiFi, thus having a longer processing time when using VHO [64] than with HHO and having problems with superfluous user numbers in the RF spectrum that cause low throughput. This makes decision-making a crucial component in HWLNets. Users in the light spectrum that encounter temporary light blockades do not need to be switched to RF, whereas fast moving users would benefit more from RF than from light spectrum. A possible solution to this problem is a handover method based on fuzzy logic [65], which takes into account user’s speed, data rate, and channel state information to make decisions. Blockages are a major impact on any network, especially LiFi, so another method was proposed in [66] that uses statistical data to optimize the handover process but has excessive computational complexity. Using artificial neural networks, we can improve a network throughput by 50% when the parameters for the decision are set on the user’s speed and the locations of APs.

### 5.4. Load Balancing

In the RF domain, load balancing is a method to distribute user’s sessions in overlapping APs. Load balancing optimizes resource employment, maximizes throughput, and reduces response time and network congestion. Load balancing happens only when geographically farthest users of an AP demand high traffic loads on neighbouring APs. If the user’s data rate requests are distributed through the network in a rectangular form, load balancing is not necessary. Load balancing techniques can be categorized [67] into:

- user-based;
- AP-based.

In the user-based technique, APs are selected depending on user’s interest, thus optimization of network performance is difficult, if not impossible to obtain. Another technique uses a central control to obtain network-wide load balancing.

**Figure 11:**
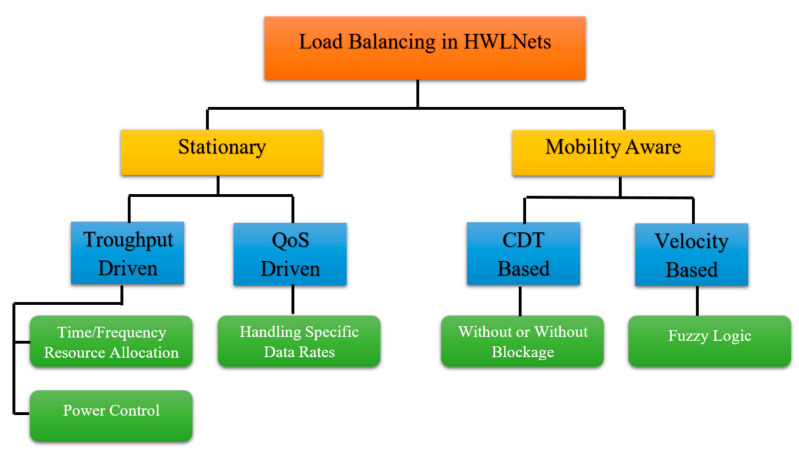
Load balancing models in hybrid LiFi–WiFi networks.

Imposing load balancing in hybrid networks is difficult due to coverage of LiFi APs and WiFi that overlap and the low system capacity of WiFi regarding LiFi [8]. Traffic overload in hybrid environments is unavoidable even with excellent geo-distribution of user demands on WiFi APs. New approaches such as cell range expansion, relaxed optimization, and game theory Markov decision process [68] minimize this problem but do not answer important constraints in HWLNets, that is user mobility, because of small coverage of LiFi transmitters. Load balancing in HWLNets can be divided into two groups, as seen in Figure 11:

- stationary;
- mobility aware.

Both use different algorithms to achieve optimized load balancing but mobility aware also takes into account user movement due to different factors.

A stationary channel has time duration over which the channel impulse response is considered to be not varying, thus load balancing has to compensate channel quality with resource availability. A method for realizing proportional fairness with the use of centralized and distributed resource algorithms to solve load balancing problem was used in [69]. Another method for QoS with mixed-integer non-linear programming problem was proposed [70]. Proposed methods have nondeterministic polynomial time complexity, which in terms requires enormous computational time and resources, thus increasing the number of APs exponentially.

Using game theory, an algorithm was proposed [71] that has multiple fairness functions including modelling blockages, different user equipment orientation, and data throughput to represent real world scenarios. Another similar method uses power allocation with focus on two key elements:

- optimize power of AP, thus maximize throughput;
- find AP that is less congested for users with low data rates and reconnect them to it.

Both of these algorithms are considered to be of autonomous optimization on the AP level. These types of algorithms, centralized or autonomous, have a high computational complexity and long processing time. HWLNets have variating channel states, especially for mobile users, which in turn limits the processing time and makes the above-mentioned methods impractical. Direct decision-making can be used to minimize the process time [72]. This method first decides which users are to be connected to WiFi, then the rest are allocated to LiFi. Lastly, this method uses statistical models of data rates and channel state information to create fuzzy-based logic and obtain optimal throughput and user fairness.

Stationary channel load balancing uses methods which calculate periodically based on movement and changes in the network. Users are moving in and out of network technologies even when they are walking inside a single LiFi domain due to constant requirement for connectivity and fairness, which leads to frequent handovers and diminishes performance.

Mobility aware load balancing takes into account user’s movements together with load balancing. The college admission model uses the moving direction of users and data rates for user predilection and for AP predilection, the total data rate is used [73]. In this method, the user’s trajectory is known in order to make a calculation, which is almost impossible in real word scenarios. Another study [43] used dynamic iterative load balancing with AP and resource allocation, thus improving data rates and minimizing handover overheads. Using cell dwell time, a method is formed to measure handover costs [74] without channel state information, which is best for varying channels. Modification of this design was proposed in [66] with the addition of blockages, thus reducing its obstructive influence.

Fuzzy logic can be used to balance traffic loads in a hybrid network [65]. This decision-making system can reduce the complexity of the above-mentioned systems relying only on velocity of movement, SINR, and data rates. This comprehensive system can be additionally upgraded with the fuzzy logic method for solving unbalanced traffic loads [53] imposed by light path blockages. Both of these algorithms use predefined rules, thus lacking real-life elasticity, whereas machine learning can provide a better model for real-time applications [75]. This method can achieve better results than all of the above-mentioned iterative algorithms.

### 5.5. Summary

Load balancing and handover are closely linked in any network system.

Frequent handovers in HWLNets are due to the channel quality and resource availability in the LiFi part of the network and impose a significant problem concerning QoS. High channel quality does not equal high data rate account of resource engagement. For the handover policy, we must consider the time user spends on a single AP without being handed over to another or disconnected all together due to user movement and blockages.

Optimization can be achieved via decision-making algorithms, taking into account that a user can be connected with multiple APs for the HHO process with an emphasis on QoS and user fairness. When HHO is no longer an option and the user would benefit more with a different network technology, VHO is employed and the user is switched.

In HWLNets, user mobility is a major factor in channel variance, thus imposing load balancing. When discoursing throughput, an arrangement has to be made to consider data rate and handover rate with regard to delay, jitter, packet loss, and others. This optimization problem can be solved in many ways but with high computational complexity. Another research approach to this problem is the use of decision-making methods that result in optimization degradation.

To make load balancing effective in multi objective scenarios, we can employ intelligent control methods which rely on user speed and queue length data or we can use predefined parameters to compose a decision scheme. These methods must have low complexity and adaptiveness in order to work in real-life scenarios.

## 6. HWLNets in IoT Applications

Supporting high data requirements in 4K/8K video streaming, holographic display, and other high-speed applications in IoT is becoming more and more important. LiFi has proven to be successful in the transmission of holographic data [76]. HWLNets can be used in a vast number of applications:

- hospitals: where RF is prohibited or cannot be used due to RF interference;
- LNG ships and platforms: due to the risk of RF;
- planes and airports: again, due to the risks involving RF in planes and congestion of traffic in airports;
- stadiums: excessive number of visitors in a given moment with different data requirements;
- conference halls: stationary users with high data and throughput requirements.

Where it can at least relieve part of the resource shortage and pressure on WiFi or provide additional service in areas where it was deemed impossible.

In IoT, user applications rely on two basic functionalities: indoor positioning and physical security.

### 6.1. Indoor Positioning

Positioning can be defined by the type of system used. There are two basic categories: outdoor and indoor. The first is widely known as a form of service employed by most consumer devices for navigation, mapping, and tracking. This service relies on satellite radio navigation data sent by global positioning system (GPS) or other variations of it such as GLONASS or BeiDou. All these systems provide GPS data to a receiver with some degree of accuracy. Using L5 band, GPS can provide accurate reading within a 30-centimetre diameter [77]. For indoor positioning, these systems are far less accurate due to the physical properties of the signal passing through non-opaque objects such as reinforced concrete or multi-storey buildings. Even if the signal passes through with enough strength in indoor scenarios, accuracy is considered in centimetres unlike metres for outdoor scenarios. High indoor accuracy can only be accomplished using positioning systems that are in the same area as the users. One such method is to use short range technologies such as Bluetooth or ZigBee. Here, we focus on alternative means of positioning using LiFi–WiFi combination methods proposed in [78,79].

**Figure 12:**
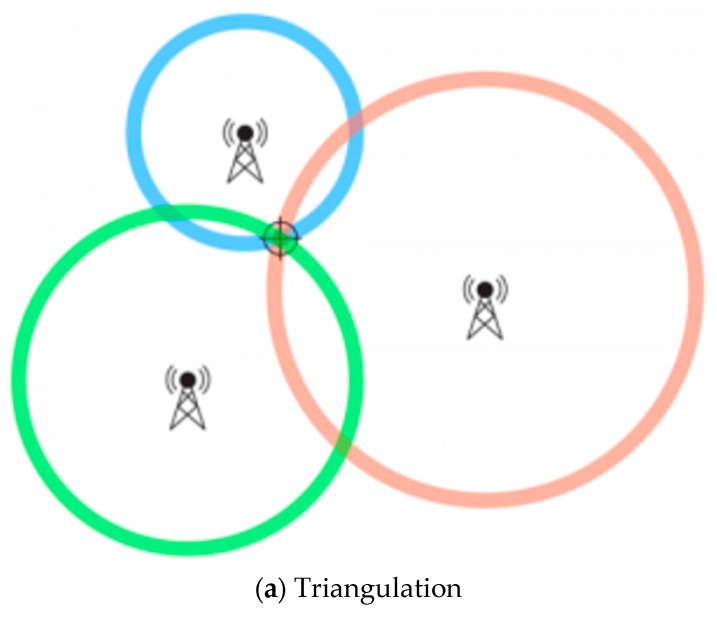

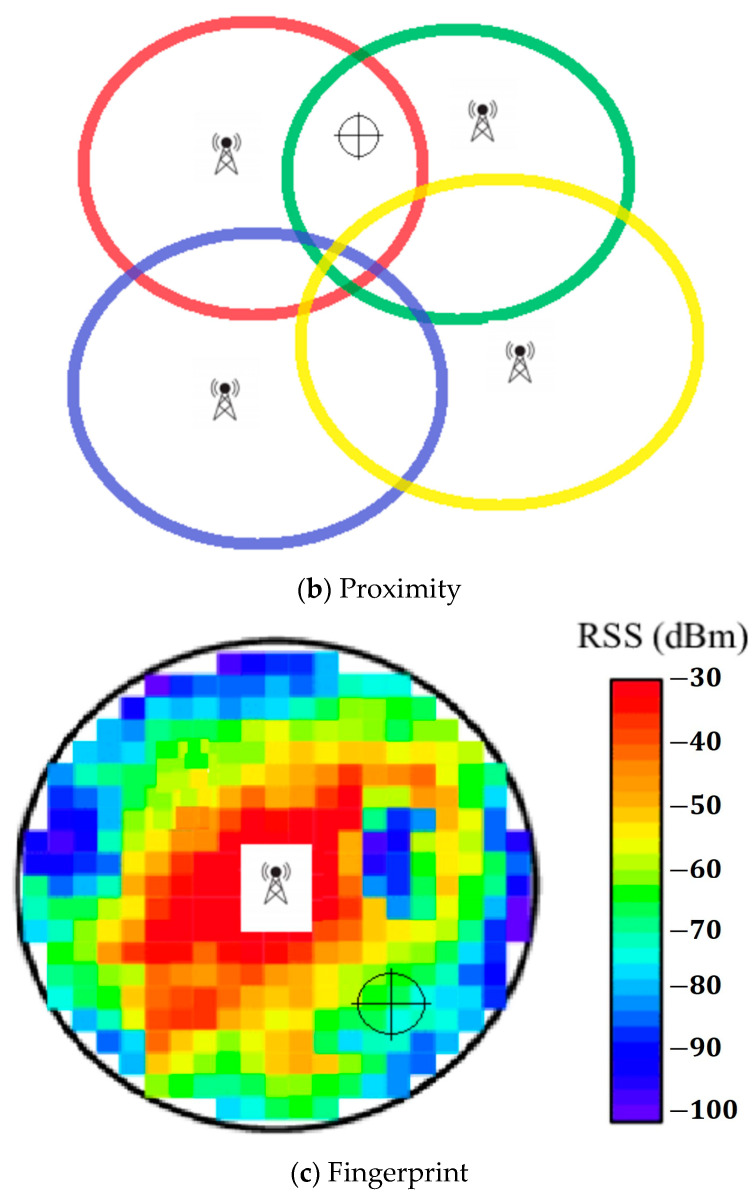
Illustration of positioning methods: (**a**) triangulation, (**b**) proximity, and (**c**) fingerprint.

Indoor positioning uses algorithms, represented in Figure 12, and information obtained from user devices or APs to calculate the exact position of a user. Triangulation algorithm uses at least three neighbouring APs to measure the distance and angle between the user’s device and AP. This method is similar to the cell tower triangulation techniques used by service provides and offers pinpoint accuracy but has a high degree of system complexity. Triangulation is usually two-dimensional but in case the LiFi user’s equipment is susceptible to orientation, as seen in Section 4, we can implement an additional AP beacon for three-dimensional triangulation [80].

Another algorithm used is proximity that connects a user’s device in regard to AP coverage area, thus having a low computational complexity. For LiFi APs that are usually densely deployed, a proximity algorithm can have decent results.

Lastly, location-dependent information algorithms such as fingerprint are suitable for indoor positioning. Using off-line radio maps and RSS, this algorithm calculates the Euclidean distance between a map and real-time data [81]. If the maps are accurate, this algorithm has a high degree of accuracy.

Indoor positioning models can determine a user’s position using information such as RSS, time of arrival, angle of arrival, or time difference of arrival. Using an RSS model of positioning, reduction of channel throughput gives an approximate distance between users and AP. This model’s major drawback is susceptibility to errors due to multipath propagations [82].

Using time of arrival to compute distance requires time sync between the user’s equipment and AP [83]. This problem can be bypassed by having multiple transmitters in the AP or receivers on user’s devices to calculate the time difference between the signals [84]. Nevertheless, time sync is needed between APs.

Angled differences in a signal that is transmitted and the nominal angle of AP are used to obtain the position in RF systems by detecting phase differences between antennas [85]. For LiFi technology, this cannot be applied, thus a new approach has been researched. Image transformation calculates distance using trigonometry of the LiFi AP’s position on a map and light image on the photodiode of the receiver [86].

### 6.2. Positioning in HWLNets

WiFi and LiFi have different positioning errors. Due to the tight deployment of LiFi and short range, it can calculate the position of users within 10 to 30 cm, whereas WiFi accuracy ranges from one to seven metres [87]. LiFi is also more cost effective due to the fact that existing lighting LED infrastructure can be used and because the user equipment in LiFi systems use angle diversity receiver to detect the device’s orientation, meaning it is easier to share data with an indoor positioning system of LiFi APs [88]. The problems in hybrid systems indoor positioning differ as a spectrum is used. Radio waves can have multipath fading when signals reach a receiver via many paths, and their relative strengths and phases change due to building materials and structures. Visual light obviously suffers from blockages by objects or even users themselves, which can influence connectivity.

In the hybrid system, one spectrum can supplement the other using its strengths where they can apply. Research [89] was done using LiFi and ZigBee with a proximity position algorithm but had low accuracy. The next research proposed a two-stage model [90] that uses LiFi to establish a possible area where a user device can be found, then with radio frequency RSS pinpoints the exact location. The error margin of this system is 20 cm. Finally, a two-stage system can be used inversely for RF to locate the approximate area, and then the nearest LiFi AP can calculate the position of the user device [91] with an error of 5.8 cm.

### 6.3. Security

In wireless communications a major security concern is the introduction of an eavesdropper into the channel. Types of man in the middle attacks reconnect users to malicious networks or use specialized programs to read unencrypted data. The use of virtual private networks solves this problem to some extent. These security barriers are made on a network or application level. Therefore, researchers are focused on physical layer security. Secure key generation uses the existing random distribution of channels (RSS) [92] to guarantee secure key distribution. In securing data transmission, we can enlarge the SINR difference in an eavesdropper’s link in contrast to the legitimate users by reducing RSS or increasing noise/interference [93].

Reducing transmission power via optimization of resource allocation and interference alignment for legitimate users thus will impair the possibility of eavesdropping.

Adding artificial noise to the sub space of channels is also an option, so that legitimate users are not affected by this addition and eavesdropper’s SINR performance is diminished. The above-mentioned models with channel coding can definitely boost security [94].

LiFi physical properties have numerous security effects, for example:

- light does not traverse outside of rooms or buildings, thus making it difficult for an outside attacker to get access to the channel;
- LiFi short range due to light properties means eavesdroppers have to be very close in order to spy;
- field of view and line of sight are imperative in VLC with 80% of the signal being in direct LoS, thus making eavesdropping minimal due to limited resources.

Researchers analysed the security performance of LiFi systems [95], proving that the hexagonal positioning of LiFi AP has the highest security capabilities and matrix slightly worse.

Quantum key distribution enables photon encoding in zero or one status or both at the same time using polarization. Quantum mechanics, such as a no-cloning principle, enables the generation of random keys between two users. Such a system was proposed in [96] using small handheld devices with an encrypted data rate of 30 kb/s and a length of fifty centimetres.

A number of studies have shown that HWLNets require less power while performing network security tasks than standalone ones [97]. Research on dual hop HWLNets have shown that LiFi signal energy can be used to relay data in the RF spectrum [98] with additional research on finding minimum transmission power that has enough security.

Another approach proposed an algorithm based on the Observe, Orient, Decide, Act (OODA) and Cyber Kill Chain (CKC) strategies to annul impersonation attacks on networks. The researchers used the RF device identification system in combination with the SDN architecture to achieve conformance with the IEEE 802.11 standard, resulting in the detection of 97.5% of attacks on the network in real-time scenarios [99]. This model is used in hybrid networks and improves security in the WiFi part, leading to an overall better performance of the whole network.

### 6.4. Summary

Hybrid networks can provide better services in terms of positioning and network security than standalone networks. For instance, for slow moving users, LiFi can provide better positioning accuracy but if the users are moving faster, the channel degrades rapidly and WiFi should be used instead. Security concerns on the physical layer are mitigated while using VLC spectrum because of its natural properties but to a certain extent because light is susceptible to connectivity issues due to numerous activities. For a secure and stable network, one must employ both spectrums to solve current issues such as using quantum key distribution to encrypt data at low rates, then send it through WiFi for fast moving users or LiFi for slow ones. A same network could use radio frequency for locating users with access rights to a network, then if they are near LiFi AP, they could be precisely pinpointed. Possibilities for these applications in HWLNets are numerous and have not been fully researched.

## 7. Implementing HWLNets

Most studies that have been conducted on HWLNets were focused on solving problems within two different network systems, LiFi and WiFi. To enable total integration, we must look at this hybrid network as one entity with both WiFi and LiFi working in parallel to avoid vertical handover, which is a root to major complications and restrictions in terms of network performance. In order to lift this, we must integrate the two technologies across network layers. This section provides a brief overview of LiFi–WiFi integration in multiple network layers.

### 7.1. Physical Layer Integration

LiFi and WiFi components can be integrated on the same platform, making a universal AP. This can be achieved by integrating front end electronics on the same board due to the fact that they share a lot of components such as power amplifiers, converters, etc. Already RF spectrum devices exist that use a reconfigurable baseband filter, which allows to serve as WiFi or cellular AP [100].

LiFi uses a different antenna technology, so implementation would prove to be difficult and a possible device might be substantial in terms of dimension compared to a standalone AP. For synchronized signal processing of both bands, signals have to be converted into different frequencies, thus impairing gain and adding noise to the channel.

Pulse modulation and OOK are the best suited modulation types for VLC with data rates up to 100 Mbps. Both are single-carrier and have low peak to average power ratio with modest data rates. In contrast, DCO-OFDM has high peak to average power ratio but offers the highest data rates. The types of LED used commercially have narrow FOV, thus have limited bandwidth. On the other hand, photo diodes in user’s equipment are vulnerable to positioning and changes in environment, especially a high volume of external light, e.g., sun light. Constructing a viable hybrid AP must take into account the compensation between spectral and energy efficiency and the circuit integration of the two different technologies.

### 7.2. Network Layer Integration

Sending packets simultaneously in hybrid networks is the goal of HWLNets researchers worldwide. This would render HWLNets biggest problem that is vertical handover. For standalone networks, TCP protocol is used to provide transmission to users in any given time by a single AP. In contrast, multipath transmission control protocol (MPTCP) [101] that is being developed allows the connection to use several paths simultaneously, thus for TCP, multipath uses sub flow system of multi-TCP connections (paths from one host to another) that are identified during the TCP three-way handshake phase. It contains data ACK and sequence mapping, which allow MPTCP to receive data from multiple sub flows in the original order sent. This revolutionary protocol can help HWLNets to avoid vertical handovers and improve on resource allocation with the use of multiple APs instead of a single one.

The above-mentioned method solves VHO problems for hybrid networks but due to the mobility of users and the short range of LiFi APs, frequent HHO still impair on HWLNets overall network performance [102]. To solve frequent HHO, researchers have developed models for cell deployment optimization [10]. Most commonly used are matrix and hexagon. In order to diminish HHO, cell deployment has to account for location, density, output power of LED transmitters, as well as WiFi APs signal and user behaviour. The latter is the most difficult to predict. Some approaches [59] have solved parts of this challenging issue but a real-time model for user’s behaviour representation is yet to be found.

The implementation of network management poses another key research objective. Management must overcome AP density and topology as well as the mobility of users, especially faster ones. This combined with the fact that changes in user equipment orientation have a massive impact on connection speed and data throughput makes this problem extremely complex in terms of processing time. Some studies [44] have shown that machine learning can improve optimization problems but collecting real-time data and processing is still a major complication.

### 7.3. Application Layer Integration

Different applications impose various data rate and latency requirements, which require high flexibility in networks. HWLNets are ideal for this due to their natural coherence between LiFi and WiFi. Network convergence in a hybrid environment can be accomplished over all layers, thus increasing its reliability, security, and consistency. Convergence for standalone networks is well examined in studies [103], however for hybrid networks, novel approaches are yet to be determined. One of the challenges faced by HWLNets is ensuring a smooth handover between the two technologies. Application layer integration plays a crucial role in addressing these challenges. This integration can be achieved through the use of SDN and network function virtualization (NFV) technologies [104]. In this model, SDN allows for centralized network control, which can be used to manage the handover process between LiFi and WiFi. NFV, on the other hand, enables the virtualization of network functions, allowing for the creation of flexible and scalable network architecture.

The use of application layer integration in HWLNets also facilitates the integration of IoT devices such as autonomous delivery vehicles in a factory or warehouse using the above-mentioned integration techniques with a higher degree of security and lower implementation cost. VLC spectrum provides additional protection at low power consumption, thus making it ideal for autonomous devices such as delivery vehicles [102]. HWLNets can support a high number of devices with widespread connectivity and resiliency, which is important for IoT devices. Another possibility is to implement HWLNets in specialized environments such as factories or mines where LiFi APs with the use of SDN applications can supplement WiFi APs in areas with low or impossible coverage. Future research in this field includes finding optimal resource description, compatible MAC protocol, prioritized packet routing, and usage of machine learning to realize intelligent network management.

## 8. Conclusions

Congestion in radio frequency spectrums has motivated researchers to develop new technologies and approaches. One such technology is LiFi, a cutting-edge network design that uses VLC with a nearly unlimited spectrum to provide high data rates with unopposed security due to the natural physical property of light. Companies such as pureLiFi, Oledcomm, and Signify have made commercially available kits for standalone LiFi implementation. However, due to constraints in LiFi transmission such as frequent handovers due to channel blockages from external light sources or opaque objects, which can cause frequent handovers or total loss of connection, in recent years, researchers have focused on hybrid network technologies as merging of LiFi with WiFi technology can mitigate these problems. Thus, introducing HWLNets can provide an improved network performance and reliability while maintaining high data rates and throughput. Moreover, data demanding applications and IoT would benefit from hybrid network implementations. Therefore, ongoing research focuses on the optimization and mathematical modelling of user mobility that immensely constrain HWLNets. Future directions could include significant hardware integration upgrades to develop a hybrid LiFi–WiFi AP, improve cell deployment optimization, and involve machine learning to enable intelligent network management.

This paper provided an overview of HWLNets framework design with special attention on cell deployment of LiFi APs, modulation schemes, and the design of backhaul devices. The performance metrics such as SINR, spectral efficiency, as well as the achieved QoS are discussed in regard to HWLNets and with comparison with standalone networks. User behaviour modelling was introduced with regard to device orientation, movement, and blockages. An overview of the handover models and load balancing is given with a focus on user mobility and algorithms addressing key issues such as computational complexity and long processing time. Finally, possible implementations of HWLNets in IoT and various applications are given with latest advancements in TCP technology that will likely solve VHO issues.

## Data Availability

Not applicable.
